# Two Novel Low-Bandgap Copolymers Based on Indacenodithiophene/Indacenodithienothiophene and Benzothiadiazole Dicarboxylic Imide: Structural Design and DFT/TD-DFT Investigation

**DOI:** 10.3390/polym17152050

**Published:** 2025-07-27

**Authors:** Bakhet A. Alqurashy, Ary R. Murad, Wael H. Alsaedi, Bader M. Altayeb, Shaaban A. Elroby, Abdesslem Jedidi

**Affiliations:** 1 Basic Science and Technologies Department, Applied College, Taibah University, Madina 42353, Saudi Arabia; 2 Department of Chemistry, College of Science, Charmo University, Chamchamal, Sulaymaniyah 46023, Iraq; 3 Department of Chemistry, Faculty of Science, Taibah University, Madina 42353, Saudi Arabia; 4 Department of Chemistry, Faculty of Science, King Abdulaziz University, Jeddah 21589, Saudi Arabia

**Keywords:** D–A conjugated copolymer, indacenodithiophene, indacenodithienothiophene, benzothiadiazole dicarboxylic imide, DFT

## Abstract

In the present study, two novel donor–acceptor (D–A) conjugated copolymers, PIDTBDI and PIDTTBDI, were successfully synthesized via Stille coupling polymerization. These alternating copolymers incorporate indacenodithiophene and indacenodithienothiophene as donor units, coupled with benzothiadiazole dicarboxylic imide as the electron-deficient acceptor unit. The influence of extended conjugation on the structural, optical, thermal, and electrochemical properties of the copolymers was systematically investigated and confirmed by density functional theory (DFT). XRD analysis confirmed that both polymers are amorphous. Thermogravimetric analysis revealed that both materials possess excellent thermal stability, with decomposition temperatures exceeding 270 °C. The theoretical and experimental values of the energy gap confirmed the thermal stability of the studied polymers. The molecular weight was determined to be 10,673 Da for PIDTBDI and 7149 Da for PIDTTBDI. Despite the variation in molecular weight, both copolymers exhibited comparable optical and electrochemical bandgaps of approximately 1.57 and 1.69 eV, respectively. Electrochemical measurements showed that PIDTBDI has a HOMO energy level of −5.30 eV and a LUMO level of −3.61 eV, while PIDTTBDI displays HOMO and LUMO levels of −5.28 eV and −3.59 eV, respectively. These results indicate that minor structural differences can considerably affect the electronic characteristics of the polymers, thus altering their overall efficacy in solar cell applications.

## 1. Introduction

The escalating global population drives up energy requirements, but our existing energy production remains heavily dependent on finite fossil fuel resources, which are being depleted at an alarming rate. Additionally, fossil fuels influence human health and create environmental issues by discharging greenhouse gases and other air pollutants [1,2,3,4]. In response, renewable energy is more feasible, workable, and eco-friendly in nature, and it is gradually replacing other energy sources [5]. Solar cells leverage the photovoltaic effect to transform sunlight into clean energy, positioning them as a pivotal technology for a sustainable future [6,7]. Lately, polymer solar cells (PSCs) have been the active research field among academia and industry stakeholders, primarily attributable to their attractive features, including device flexibility, reduced production costs, lightweight construction, and solution processability [8,9,10,11,12,13]. These distinctive properties position PSCs as promising candidates for next-generation solar technologies, particularly in applications where conventional silicon-based photovoltaics fall short [14]. Unlike brittle inorganic technologies, PSCs offer inherent mechanical flexibility, making them well-suited for flexible organic solar cells, which makes them ideal for wearable electronic devices, building-integrated photovoltaics, and portable power systems [15,16,17]. Their solution processability enables scalable, low-cost fabrication through techniques such as spin-coating and roll-to-roll printing [18,19,20]. Additionally, PSCs are extremely lightweight, typically under 0.5 kg/m^2^, due to ultrathin active layers (<2 μm) [21,22]. The highest-performing PSCs employ a bulk-heterojunction (BHJ) architecture, featuring a photoactive layer that blends a conjugated polymer donor with a fullerene derivative (PC_61_BM/PC_71_BM) or non-fullerene small molecule acceptor (NFA) [23,24,25]. The nanoscale phase separation enables the creation of a BHJ morphology with an extensive donor-acceptor interface, promoting excellent properties like exciton dissociation and bicontinuous pathways for charge carrier transport [26].

Generally, conjugated polymers should possess five central characteristics: (i) narrow optical bandgap (generally between 1.2 and 1.8 eV) and high extinction coefficients (>10^5^ cm^−1^) [27,28], enabling broad absorption of visible and near-infrared sunlight; (ii) optimized frontier molecular orbitals, specifically highest occupied molecular orbital (HOMO) and lowest unoccupied molecular orbital (LUMO) levels, which are essential to ensure a high open-circuit voltage (V_OC_) and facilitating a good exciton dissociation through a favorable energy offset; (iii) good solubility in frequently used organic solvents to facilitate solution processing and miscibility with materials of acceptor; (iv) high hole mobility (over 10^−3^ cm^2^ V^−1^ s^−1^) to enhance charge transport and minimize recombination. Notably, Kang et al. [29] reported an exceptionally high hole mobility of 12 cm^2^ V^−1^ s^−1^ and (v) high molecular weight (>10,000 Da), which improves film formation and chain entanglement [30,31,32]. Deshmukh et al. [33] recently reported a record number-average molecular weight (M_n_) of 167 kDa for a fluorinated PNDITF4T acceptor polymer. To date, donor (D)–acceptor (A) polymerization with electron-rich donors and electron-deficient acceptors is one of the most effective methods to meet these criteria [34,35]. By leveraging internal charge transfer (ICT) between the donor and the acceptor units, researchers can manipulate polymers’ properties like charge carrier mobility and optoelectronic properties through the strategic selection of D–A pairs [36]. After decades of effort by researchers worldwide, power conversion efficiencies (PCEs) of PSCs based on donor and acceptor strategy have significantly increased, reaching up to 19%, approaching the requirement of industrial applications [37]. Indacenodithiophene (IDT) and its derivatives show promise as donor blocks in conjugated copolymers for organic electronics [38,39]. IDT is a widespread ladder-type donor unit with five-membered multifused rings. This donor features a unique π-conjugated framework, where two thiophene and one benzene ring are fused together, forming a planar structure with two bridging atoms, providing numerous benefits, including enhanced coplanarity, longer conjugation along the polymer backbone, promoting intermolecular π-π interaction, and reducing the reorganization energy [40]. The bridging atoms also enable the attachment of aryl or alkyl side chains, which can significantly modulate direct self-assembly and the morphology of a thin film, improve its solubility, and regulate aggregation, ultimately tuning the polymer’s energy levels [41]. IDT is believed to be a promising donor unit to construct D–A copolymers for PSCs [42,43]. Chen and his coworkers synthesized a copolymer (PIDTBT) that is based on IDT and 2,1,3-benzothiadiazole (BT) as the acceptor [44]. It has an optical bandgap (E_g_) of 1.75 eV, having a high PCE value equal to 6.4%, upon mixing with PC_71_BM. Soon after, two new copolymers (PIDT-diphQ and PIDT-phanQ) were synthesized by Jen et al., using IDT as D and quinoxaline as an A [45]. PIDT-phanQ has a lower E_g_ (1.67 eV) compared to PIDT-diphQ (1.81 eV). Consequently, the **PIDT-phanQ:PC_71_BM** BHJ device showed a higher value of PCE than the **PIDT-diphQ:PC_71_BM**: 6.24% for the former and 5.69% for the latter [45]. **PIDTDTQ: PC_71_BM** (1:4) blends exhibited a remarkable PCE of 7.5% by flanking two thiophene units between quinoxaline and IDT, in which octyloxy side chains are located on the meta positions of quinoxaline [46]. Recently, IDT was copolymerized with 5,6-dioctyloxy-2,1,3-benzothiadiazole (BTO) as an acceptor with two thiophene or thienothiophene as the spacers to form two new alternating copolymers (PIDTBTO-T and PIDTBTO-TT) [38]. PIDTBTO-TT based PSCs showed an 8.15% increase in the value of PCE in single junction and 11.15% in tandem solar cells [38]. Recently, Iain McCulloch et al. constructed a D–A copolymer (PIDT_C16_-BT), which has a linear hexadecyl side chain that demonstrated a high hole mobility of 3.6 cm^2^ V^−1^ s^−1^, although their copolymers did not show long-range structural order [47]. The promising mobility in a material, having a restricted crystallinity, is because of the outstanding resistance conformation of the backbone towards the side chain disorder [48]. In the last decade, IDT and its derivatives can also be used for developing and synthesizing various types of non-fullerene acceptors (NFAs) employed in efficient PSCs [49,50,51].

The compound 2,1,3-benzothiadiazole (BT) and its derivatives, such as dithienyl benzothiadiazole (DTBT), dialkoxy benzothiadiazole [(OR)_2_BT], and difluoro benzothiadiazole (DFBT), are extensively employed in electron-accepting repeat units and are being used to make low-bandgap D–A copolymers [52,53,54]. A novel class of benzothiadiazole-based copolymers (BDIs) was reported by Wang and co-workers, which integrated a dicarboxylic imide at the 5,6-positions of the BDI [55]. For instance, to enhance solubility and facilitate the processability of the resulting polymer, different solubilizing groups can be added to the *N* atom of the dicarboxylic imide moiety [56]. The BDI acceptor has a stronger electron-withdrawing nature, which typically shows a lower value of the bandgap and a deeper HOMO value [57]. This is ascribed to the high V_OC_ observed in the fabricated BHJ solar cell [58]. Furthermore, BDI-based copolymers showed an impressive PCE of up to 8% when fabricated into a BHJ photovoltaic cell with no addition of any solvent or annealing process [59]. Moreover, BDI-containing copolymers exhibited promising hole mobilities to the value of 0.70 cm^2^ V^−1^ s^−1^ when transformed into organic field effect transistors [60].

This study entails the preparation of two novel D–A copolymers, PIDTBDI and PIDTTBDI, constructed upon indacenodithiophene/indacenodithienothiophene and benzothiadiazole dicarboxylic imide units. These copolymers have been synthesized, characterized, and confirmed by theoretical density functional theory (DFT) calculations. They have shown promising properties that could be used in different technologies, especially PSCs.

## 2. Experimental Section

### 2.1. Materials

Aldrich and Alfa Aesar’s materials were purchased for this work and used without any purification, unless otherwise mentioned. Argon was used as an inert gas, and sodium was used for drying and distilling toluene. The compounds 4,4,9,9-tetrakis(4-hexylphenyl)-4,9-dihydro-s-indaceno [1,2-b:5,6-b’]dithiophene-2,7-diyl)bis(trimethylstannane) (M1), 6,6,12,12-tetrakis(4-hexylphenyl)-indacenodithieno [3,2-b]thiophene-2,8-diyl)bis(trimethylstannane) (M2), 4,7-di(5-bromo-thien-2-yl)-2,1,3-benzothiadiazole-5,6-*N*-(3,7-dimethyloctyl)dicarboxylic imide (M3) were synthesized as mentioned in the previous studies [61,62,63]. Please see the following references for detailed information regarding instruments being used for measurements [64,65].

### 2.2. Preparation of Polymers

#### 2.2.1. Synthesis of PIDTBDI

A mixture of M1 (0.129 g, 1.045 × 10^−4^ mol) with M3 (0.070 g, 1.045 × 10^−4^ mol), tris(dibenzylideneacetone)dipalladium(0) Pd_2_(dba)_3_ (0.003 g, 3.28 × 10^−3^ mmol) and tri(*o*-toly)phosphine [P(*o*-tol)_3_] (0.002 g, 6.57 × 10^−3^ mmol) was initially degassed with argon, and then 6 mL of toluene was mixed. For 55 h, this mixture was heated to 110 °C and then precipitated in methanol. The polymer was then filtered and extracted using Soxhlet extraction with methanol, acetone, hexane, and chloroform sequentially. Subsequently, polymer was attained by precipitating the chloroform fraction in methanol, which is dark green in color and solid in nature (0.095 g, 6.583 × 10^−5^ mol, 62% yield). ^1^H NMR (C_2_D_2_Cl_4_, δ): 8.11 (br, 2H), 7.85 (br, 2H), 7.60–6.80 (m, br, 20H), 3.78 (s, br, 2H), 2.58 (s, br, 8H), 1.95–0.63 (bm, 63H). GPC results: Mn = 4334 Da; Mw = 10,673 Da; PDI = 2.46.

#### 2.2.2. Synthesis of PIDTTBDI

PIDTTBDI was synthesized using the same procedure as PIDTBDI. A mixture of M2 (0.140 g, 1.045 × 10^−4^ mol) with M3 (0.070 g, 1.045 × 10^−4^ mol), Pd_2_(dba)_3_ (0.003 g, 3.28 × 10^−3^ mmol), and P(*o*-tol)_3_ (0.002 g, 6.57 × 10^−3^ mmol) were degassed with argon, and then 6 mL of toluene was mixed. In this case, the whole sample was also heated for 55 h until it reached 110 °C. The obtained polymer was again dark green in color and solid in nature (0.062 g, 3.986 × 10^−5^ mol, 38% yield). ^1^H NMR (C_2_D_2_Cl_4_, δ): 8.08 (br, 2H), 7.84 (br, 2H), 7.70–6.94 (m, br, 20H), 3.78 (s, br, 2H), 2.60 (s, br, 8H), 2.01–0.63 (bm, 63H). GPC results: Mn = 6236 Da; Mw = 7149 Da; PDI = 1.14.

### 2.3. Computational Details

All calculations were carried out using the G09 program package [66]. The possible conformers of the studied molecules were optimized at the B3LYP/6-311++G ** level of theory [67,68,69]. Frequency calculations were performed to confirm whether the structures corresponded to global minima or transition states on the potential energy surface, identified by the presence of zero or one imaginary frequency, respectively. The UV–Vis absorption spectra of PIDTBDI and PIDTTBDI were simulated using the Time-Dependent Density Functional Theory (TD-DFT) method [70] at the B3LYP/6-311++G ** level of theory. These calculations aimed to investigate electronic properties, including HOMO-LUMO energies, dipole moments, absorption wavelengths, and oscillator strengths.

The Non-Covalent Interaction (NCI) analysis provides an index based on electron density and its derivatives, enabling the identification of non-covalent interactions [71]. The NCI index is derived from a 2D plot of the reduced density gradient (s) versus the electron density (ρ) as shown in Equation (1):

(1) $$s=\frac{1}{{2({3\pi }^{2})}^{\frac{1}{3}}}\frac{\left|\nabla \rho \right|}{\rho^{\frac{4}{3}}}$$

The Multiwfn Software version 3.7 was used to generate plots of the electron density (ρ) and reduced density gradient (RDG) for PIDTBDI and PIDTTBDI [72].

## 3. Results and Discussion

### 3.1. Polymer Synthesis

**Scheme 1:**
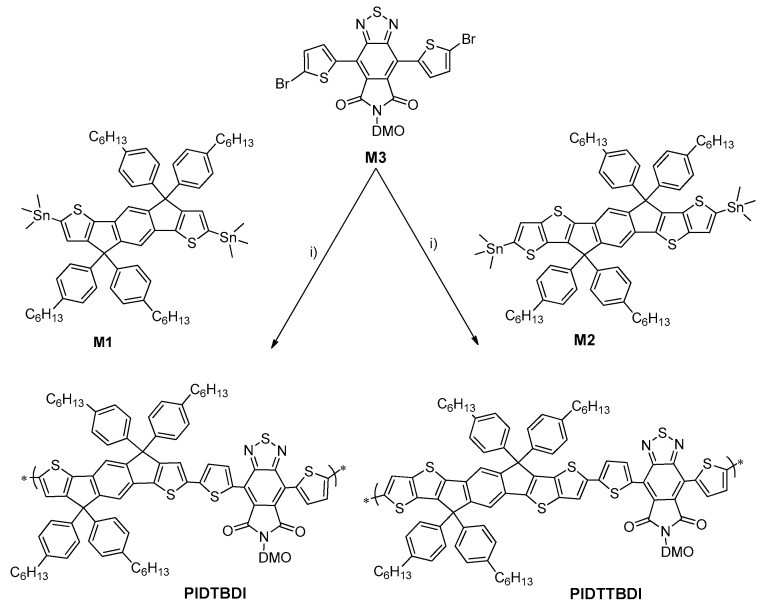
The synthesis of PIDTBDI and PIDTTBDI via the Stille cross-coupling reaction. Reagents and conditions: (i) Pd_2_(dba)_3_, P(o-tol)_3_, toluene, 110 °C, 55 h.

PIDTBDI and PIDTTBDI copolymers were synthesized via Stille polycondensation. In this polymerization, bis-stannyl monomers (M1 and M2) and the dibromo monomer (M3) were employed, with Pd_2_(dba)_3_ and P(o-tol)_3_ serving as the catalyst and ligand, respectively, in a toluene solution, as illustrated in Scheme 1. This polymerization process was performed for 55 h in an inert argon gas, with the temperature maintained around 110 °C. Polymers were fractionated using methanol, acetone, hexane, and chloroform via Soxhlet extraction. After fractionating, the final solid product, having a dark green color, was obtained by using methanol to precipitate the chloroform solution. ^1^H NMR spectroscopy was used for chemical structure analysis (see Supporting Information). The molecular weight distribution was characterized using gel permeation chromatography (GPC), using 1,2,4-trichlorobenzene (TCB) as the eluent, keeping a 140 °C temperature and polystyrene standards for calibration (see Supporting Information). PIDTBDI and PIDTTBDI displayed *M_n_* of 4334 and 6236 Da, respectively, having polydispersity indices (PDI) of 2.46 and 1.14. Table 1 summarizes these values. Both copolymers exhibited good solubility in tetrahydrofuran (THF), chlorobenzene (CB), 1,2-dichlorobenzene (DCB), and as well as in chloroform (CHCl_3_).

### 3.2. Thermal Properties and Powder X-Ray Diffraction Studies

**Figure 1:**
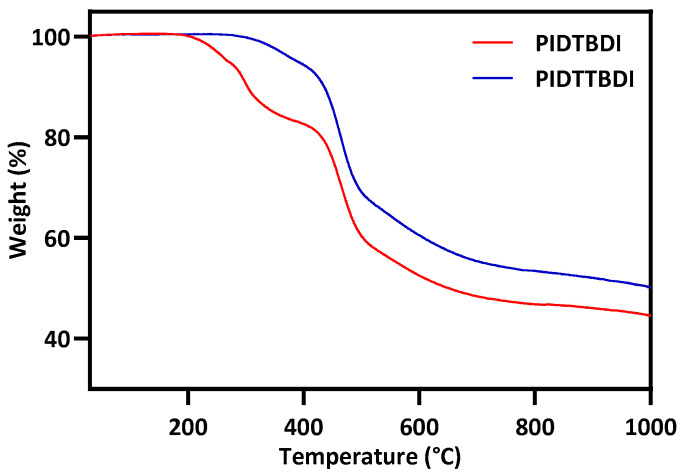
TGA behavior of the PIDTBDI and PIDTTBDI copolymers.

Thermogravimetric analysis (TGA), as shown in Figure 1, depicted that PIDTBDI and PIDTTBDI exhibit promising thermal stability, with only 5% weight-loss degradation occurring at 270 and 390 °C, respectively (Table 1). This was ascribed to the removal of 3,7-dimethyloctyl and four hexyl phenyl chains from the polymer backbone.

**Figure 2:**
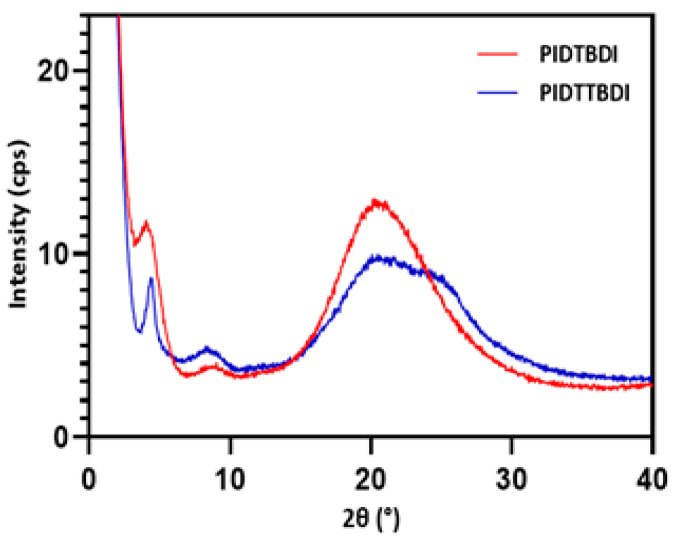
Powder XRD patterns of the PIDTBDI and PIDTTBDI.

The powder X-ray diffraction (XRD) patterns showed multiple diffraction peaks between 4.19° and 22.35° for both copolymers, as depicted in Figure 2. The first peak for PIDTBDI is at 2θ = 4.19° and, for PIDTTBDI, it appeared at 2θ = 4.55°, reflecting the spacing between polymer backbones. Broad peaks around 2θ = 9° are observed for both polymers, indicating the distances between the alkyl side chains. Additionally, two broad diffraction peaks at 2θ = 20.51° and 22.35° for PIDTBDI and PIDTTBDI, respectively, correspond to the π−π stacking distances. Both polymers exhibit an amorphous nature, as indicated by the absence of sharp peaks in the XRD patterns, which is consistent with other amorphous conjugated D–A copolymers [73,74]. The observed amorphous character suggests limited long-range molecular ordering, which may hinder exciton dissociation and charge carrier mobility but might promote finer phase separation when blended with fullerene/non-fullerene acceptors.

To evaluate crystallite size for PIDTBDI and PIDTTBDI, the Williamson–Hall (W-H) method was employed [75]. This method considers both crystallite size and strain contributions to XRD peak broadening, unlike the Scherrer equation, which only accounts for size [76,77,78]. The W–H equation is given by Equation (2):

(2) βcosθ=kλ/D+4ϵsinθ

where *β* is the full width at half maximum (FWHM) in radians, *θ* is the Bragg angle, *λ* is the X-ray wavelength, *k* is the shape factor (taken as 0.94), *D* is the crystallite size, and *ϵ* is the microstrain. The W–H analysis revealed that PIDTBDI exhibited a crystallite size of 2.10 nm, whereas PIDTTBDI showed a significantly larger size of 6.26 nm, indicating a higher degree of crystallinity in the latter. This difference may be attributed to enhanced backbone planarity and π–π stacking in PIDTTBDI, promoting more ordered domains [79]. The full analysis, including peak positions, β values, and detailed W–H calculations, is provided in the Supporting Information (Table S1 and Figure S1).

### 3.3. Optical Properties

**Figure 3:**
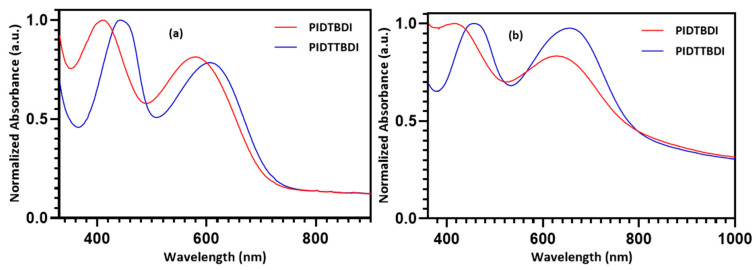
Absorption spectra of PIDTBDI and PIDTTBDI in chloroform solution (**a**) and thin films (**b**).

Figure 3a illustrates the absorption spectra in dilute chloroform solution, and Figure 3b shows thin films. The absorption maximum (λ_max_) and optical bandgaps (E_g_^opt^) are presented in Table 1**.** PIDTBDI and PIDTTBDI demonstrate two distinct absorption bands, as can be seen in Figure 3. The first band, located in the shorter wavelength region, is attributed to the π-π* transition. In contrast, the second band appears in the longer wavelength region and arises from strong intramolecular charge transfer (ICT) between the BDI as A and IDI and IDTT as D units. By extending the conjugation length from PIDTBDI, which contains IDT, to PIDTTBDI, incorporating IDTT, the absorption maxima experience a red shift in both solutions (from 580 to 625 nm) and thin film (from 605 to 657 nm), respectively. These shifts in peaks could be related to improved ICT. The increase in conjugation length of the IDTT donor unit causes these enhancements. From absorption edges (*ca*. 782 and 788 nm) of PIDTBDI and PIDTTBDI, the values of E_g_^opt^ are estimated to be 1.58 and 1.57 eV, respectively.

The optical bandgaps of both polymers were further evaluated using the absorption spectrum fitting (ASF) method derived from Tauc’s relation [80] presented in Equation (3):

(3) $${\left(\alpha hv\right)=B(hv-E_{g})}^{\gamma }$$

This method is usually applied to measure the value of electronic transitions in semiconducting materials. ASF plots based on this relation were performed for PIDTBDI and PIDTTBDI and are presented in Figure S1, with details of the methodology in the Supporting Information. The calculated bandgaps were 1.51 eV for PIDTBDI and 1.55 eV for PIDTTBDI, which are in close agreement with those estimated from the absorption onset method in Table 1.

PIDTBDI showed a narrower optical bandgap compared to PIDTBT (1.75 eV) and PIDTDTBT (1.74 eV), which are based on IDT as donor and benzothiadiazole and dithienyl benzothiadiazole as acceptor units, as reported by Chen and colleagues [81]. PIDTBDI and PIDTTBDI have significantly lower bandgaps relative to PIDT-DFBT and PIDTT-DFBT (1.78 eV) based on IDT and IDTT as donor units and a difluoro benzothiadiazole as acceptor unit, synthesized by Xu and co-workers [63]. In addition, PIDTBDI has a lower E_g_ ^opt^ around 0.1-0.2 eV than the polymers with the same donor but 2,3-diphenylquinoxaline (diphQ) and phenanthrenequnioxaline (phanQ) as acceptor units [45]. This is associated with the stronger BDI as an acceptor unit compared to BT, DTBT, DFBT, diphQ, and phanQ moieties.

### 3.4. Density Functional Theory (DFT) Analysis

**Figure 4:**
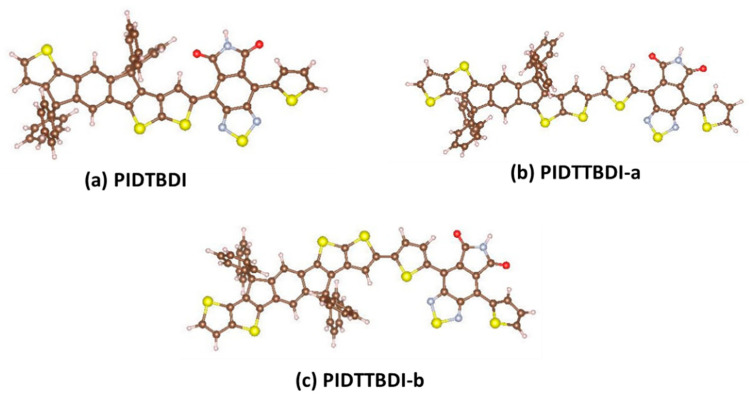
Structure of the studied compounds PIDTBDI, PIDTTBDI-a, and PIDTTBDI-b. Carbon atom in red, Nitrogen atom in grey, Sulfur atom in yellow, oxygen atom in red and hydrogen atom in pink.

The two main structures (PIDTBDI and PIDTTBDI) were modeled as shown in Figure 4, with a third structure as a rotational isomer from PIDTTBDI (named PIDTTBDI-b), which differs from PIDTTBDI-a by the rotation around the C-C bond between the two thiophene rings on the right side of the molecule.

**Figure 5:**
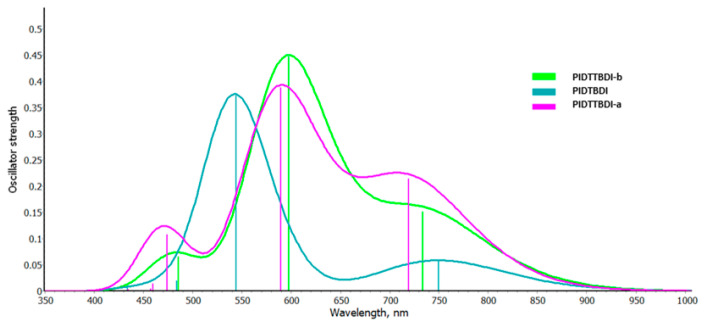
UV–Vis spectra of PIDTBDI, PIDTTBDI-a, and PIDTTBDI-b molecules in the gas phase obtained by using TDDFT/B3LYP/6-311++G** level of theory.

The absorption spectra of the three structures are shown in Figure 5, and their corresponding maximum absorption wavelengths are summarized in Table 2. Among the studied systems, PIDTBDI exhibits the shortest absorption peak at 543 nm, while PIDTTBDI-a and PIDTTBDI-b display red-shifted peaks at 589 nm and 597 nm, respectively. Notably, the absorption maximum of PIDTTBDI-b closely matches the experimental value of 605 nm. This red shift reflects a reduction in the optical bandgap, likely attributed to enhanced π-conjugation and increased structural planarity.

The introduction of the IDTT unit and its conformational variation between PIDTTBDI-a and PIDTTBDI-b play a significant role in tuning the optoelectronic properties. PIDTTBDI-b, with the longest wavelength and highest oscillator strength, appears to have the most favorable conformation for conjugation and light harvesting, making it potentially more effective in optoelectronic applications such as organic photovoltaics. When compared with experimental results in Table 1, the calculated absorption bands show a blue shift of 8–37 nm, which can be attributed to the absence of solvation effects, as the theoretical calculations were performed in the gas phase (Figure 5). Additionally, the HOMO and LUMO orbitals are localized differently in PIDTBDI and PIDTTBDI. In PIDTBDI, the frontier orbitals are spatially co-localized on the same side of the molecule, indicating weaker ICT, which may result in a shorter exciton lifetime. In contrast, PIDTTBDI shows orbital separation across opposite sides of the molecule, resulting in a significantly stronger ICT. This leads to red-shifted absorption and a reduced HOMO–LUMO energy gap (see Table 3).

The HOMO and LUMO levels, along with the energy gap, directly influence the electrochemical and thermal characteristics of the polymers. Typically, a decreased LUMO level facilitates reduction, whereas an increased HOMO level promotes oxidation. A larger energy gap suggests enhanced stability and improved resistance to thermal degradation. A larger energy gap suggests enhanced stability and improved resistance to thermal degradation. By forecasting the reactivity, stability, and potential applications of the polymers in thermal and electronic devices, these electronic elements can assist designers in developing enhanced polymer materials. The energy gap (E_g_) in Table 3 shows a decrease from PIDTBDI to PIDTTBDI, which is in agreement with experimental findings.

The energy levels of the HOMO and LUMO are significantly related to the oxidation and reduction potential of a polymer. A higher (less negative) HOMO energy indicates that the polymer is more easily oxidized (which means it exhibits a lower oxidation potential). Conversely, a lower (more negative) HOMO energy suggests that the polymer is more resistant to oxidation.

Based on the values of the energy levels in Table 3, the order of ease oxidation is PIDTTBDI-b > PIDTTBDI-a > PIDTBDI. Similarly, the LUMO energy level is directly related to the reduction potential of a polymer. The order of ease of reduction is PIDTTBDI-b > PIDTBDI > PIDTTBDI-a, depending on the LUMO energy values as presented in Table 3. This indicates that PIDTBDI is the least chemically susceptible to oxidation, whereas PIDTTBDI-b is the one that oxidizes most readily. This characteristic is crucial for uses such as organic solar cells or hole transport layers, where electron donation is vital.

The energy gap between HOMO and LUMO is a key indicator of a polymer’s thermal stability. A larger energy gap typically indicates greater thermal stability, as more energy is necessary to excite electrons or trigger bond breaking. On the other hand, a small energy gap suggests lower thermal stability. Based on energy gap values presented in Table 3, PIDTTBDI-b would be the least thermally stable of the three polymers, but PIDTBDI would have the maximum thermal stability.

**Figure 6:**
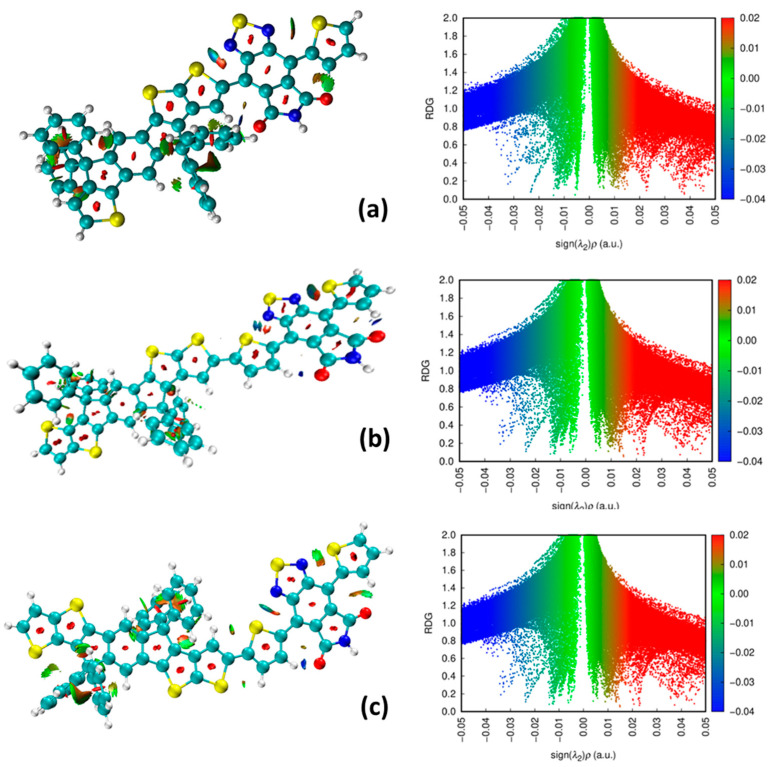
3D NCI plots (left) and 2D NCI-RDG scatter plot (right) of the three molecules (**a**) PIDTBDI (**b**) PIDTTBDI-a, and (**c**) PIDTTBDI-b. Carbon atoms in cyan, sulfur in yellow, nitrogen in blue, oxygen in red and hydrogen in white. In NCI-RDG plot, blue color refers to the attraction in negative range, red is for repulsion with positive range and green for weak interaction for values around zero.

When weak inter- or intramolecular interactions occur, there is a significant change in the RDG between interacting atoms, resulting in density critical points between the interacting fragments. Troughs appear in the s(ρ) plot, corresponding to these critical points. At low densities, the behavior of s is dominated by ρ, causing s to diverge except in regions near a density critical point, where the gradient of the electron density (∇ρ) dominates, and s approaches zero (see Figure 6).

### 3.5. Electrochemical Properties

**Figure 7:**
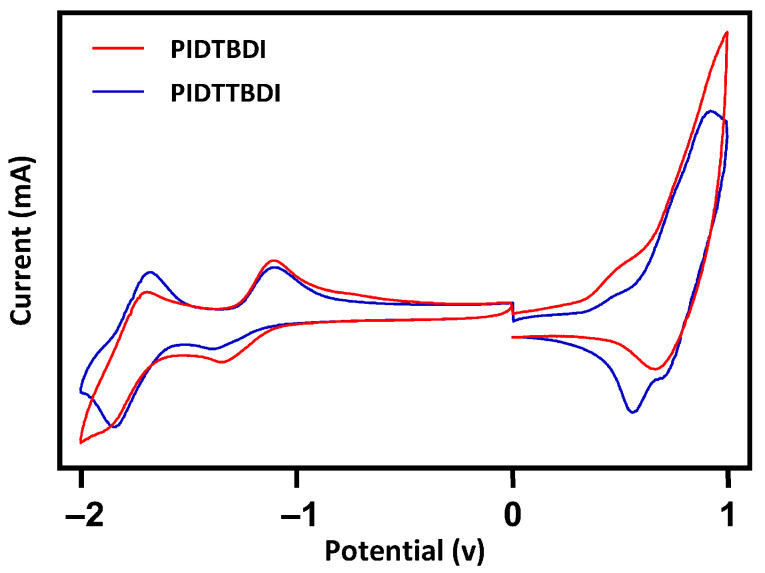
Cyclic voltammograms of PIDTBDI and PIDTTBDI.

In this investigation, cyclic voltammetry was utilized to examine frontier energy levels (HOMO and LUMO) of the two copolymers (Table 4 and Figure 7). The HOMO levels of PIDTBDI and PIDTTBDI were −5.30 and −5.28 eV, respectively, based on their onset oxidation potentials. The results indicate that the extension of conjugation from IDT to IDTT had only a minimal impact on the HOMO energy levels of the copolymers. Xu et al. reported that the HOMO level of PIDTT-DFBT (−5.30 eV) was higher in comparison to PIDT-DFBT (−5.46 eV), which was attributed to the electron-rich nature of the IDTT unit compared to IDT [63]. PIDTBDI’s HOMO level is lower than that of the PIDTDTBT alternating copolymer synthesized by Chen et al. (−5.19 eV), which utilizes the DTBT moiety instead of BDI [81]. Zhang et al. identified that the HOMO levels of PIDT-diphQ and PIDT-phanQ (−5.33 and −5.28 eV) are close to that of PIDTBDI [45]. In contrast, the HOMO level of PIDTBDI is larger than that of PIDTDTQx, which incorporates IDT as the D and dithienyl quinoxaline (DTQx) as the A, similar to the results of Guo et al. (−5.11 eV) [46]. The LUMO energy levels of PIDTBDI and PIDTTBDI were determined to be −3.61 and −3.59 eV, respectively, based on their onset reduction potentials. Notably, both copolymers exhibit nearly identical LUMO levels due to the presence of the same BDI acceptor segment, which predominantly governs the LUMO energy in conjugated polymers. Additionally, the E_g_ ^elec^ for both copolymers were calculated to be 1.69 eV.

Table 5 presents the thermal, optical, and electrochemical properties of PIDTBDI and PIDTTBDI alongside structurally related D–A copolymers reported in the literature. The selected reference polymers are based on IDT and IDTT units combined with either benzothiadiazole derivatives or thienopyrroledione (TPD) moieties. Our polymers exhibit the lowest optical bandgaps among those listed, which is beneficial for wide light absorption in PSC applications. The HOMO energy levels of PIDTBDI and PIDTTBDI indicate adequate oxidative stability while preserving compatibility with high V_oc_. Notably, PIDTTBDI demonstrates a higher thermal decomposition temperature compared with the other examples, reflecting the enhanced thermal robustness provided by the extended and more planar IDTT structure. Although photovoltaic device measurements were not performed in this work, the optoelectronic properties of PIDTBDI and PIDTTBDI are comparable to those of several high-performing copolymers, which achieved PCEs in the range of 4.4% and 12.7%. These results highlight the potential of our copolymers for efficient and stable PSCs.

**Scheme 2:**
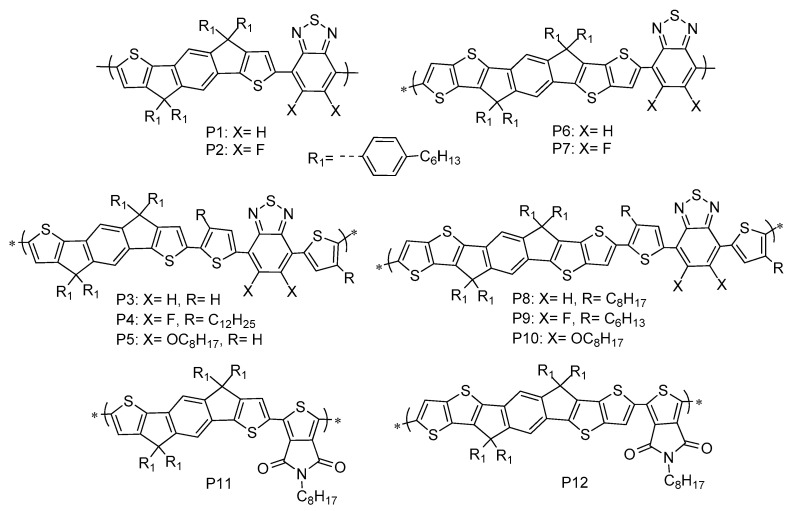
Chemical structures of IDT- and IDTT-based copolymers.

## 4. Conclusions

Two conjugated copolymers, PIDTBDI and PIDTTBDI, were successfully synthesized via Stille cross-coupling polymerization by combining indacenodithiophene (IDT) and indacenodithienothiophene (IDTT) as electron-donating units with benzothiadiazole dicarboxylic imide (BDI) as the electron-accepting unit, bridged by thiophene π-linkers. Both copolymers demonstrated excellent solubility in common organic solvents, attributed to the presence of four hexylphenyl substituents on the IDT and IDTT units and a 3,7-dimethyloctyl chain on the BDI moiety. The chemical structures were confirmed by ^1^H NMR spectroscopy, while the optoelectronic, thermal, and structural properties were investigated using UV–vis absorption spectroscopy, TGA, XRD, and CV. TGA results revealed good thermal stability, with 5% weight loss observed at 270 °C for PIDTBDI and 390 °C for PIDTTBDI. Powder XRD patterns indicated that both polymers possess predominantly amorphous structures, suggesting limited long-range ordering. GPC analysis showed that PIDTTBDI exhibits a higher *Mn* compared to PIDTBDI, likely due to the extended conjugation of the polymer backbone. Additionally, the strong ICT between the electron-rich donor units (IDT and IDTT) and the electron-deficient BDI acceptor contributes to the formation of narrow bandgap materials (~1.57 eV) with broad absorption profiles extending from 350 to approximately 782–788 nm in the solid state. These experimental results were supported by DFT and TD-DFT simulations, which showed consistent trends, with calculated absorption maxima exhibiting a blue shift of 8–37 nm. Overall, the structural and optoelectronic characteristics of PIDTBDI and PIDTTBDI highlight their potential as promising candidates for application in PSCs.

## Figures and Tables

**Table 1:** Molecular weights, polydispersity indices, thermal, and optical properties of PIDTBDI and PIDTTBDI.

Polymer	*M*_n_ (Da) ^a^	*M*_w_ (Da) ^a^	PDI	T_d_ (°C) ^b^	λ_max_ (nm)		E_g_^opt^ (eV) ^c^
					Solution	Film	
PIDTBDI	4334	10673	2.46	270	580	625	1.58
PIDTTBDI	6236	7149	1.14	390	605	657	1.57

“^a^ Measured by GPC”; ^“b^ Measured by thermogravimetric analysis”; “^c^ Optical bandgap (E_g_ ^opt^)”.

**Table 2:** Electronic absorption bands nm; corresponding oscillator strengths; natural transition orbitals (NTO) by using TD-DFT-B3LYP//6-311++G** level of theory.

Compound	λ_max (nm)_	f	Assignment	Hole	Particle
PIDTBDI	543	0.373	H-1→L	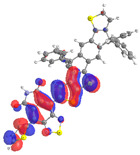	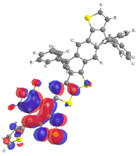
PIDTTBDI-a	589	0.388	H-1→L	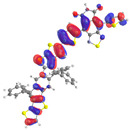	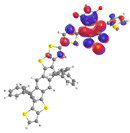
PIDTTBDI-b	597	0.447	H-1→L	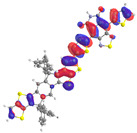	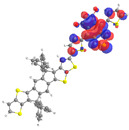

**Table 3:** The calculated HOMO, LUMO, and energy gap of the studied molecules by using B3LYP/6-311++G** level of theory.

Compound	HOMO (eV)	LUMO (eV)	Energy Gap (eV)
PIDTBDI	−5.480	−3.470	2.010
PIDTTBDI-a	−5.435	−3.460	1.975
PIDTTBDI-b	−5.431	−3.501	1.930

**Table 4:** Electrochemical properties of the polymers.

Polymer	HOMO (eV) ^a^	LUMO (eV) ^a^	E_g_ ^elec^ (eV) ^b^
PIDTBDI	−5.30	−3.61	1.69
PIDTTBDI	−5.28	−3.59	1.69

“^a^ Measured by cyclic voltammetry”; “^b^ Electrochemical bandgap (E_g_ ^elec^)”.

**Table 5:** Optical, electrochemical, and thermal properties of IDT- and IDTT-based copolymers. The chemical structures are provided in Scheme 2. The first two entries are from this study; the rest are from the literature.

Polymer	E_g opt_ (eV) ^b^	HOMO (eV) ^c^	LUMO (eV) ^c^	E_g_ ^elec^ (eV) ^d^	T_d_ (°C)	PCE (%)	Ref
PIDTBDI	1.58	−5.30	−3.61	1.69	270	-	This work
PIDTTBDI	1.57	−5.28	−3.59	1.69	390	-	
P1	1.75	−5.23	−3.52	1.71	-	6.30	[81]
P2	1.78	−5.46	−3.56	1.90		5.97	[62]
P3	1.74	−5.19	−3.49	1.70	-	4.40	[81]
P4	1.80	−5.71	−3.70	2.01	-	12.7	[82]
P5	1.89	−5.29	−3.53	1.76	335	6.05	[83]
P6	1.75	−5.75	−3.64	2.11	-	6.20	[84]
P7	1.78	−5.82	−3.71	2.11	-	6.10	[84]
P8	1.79	−5.28	−3.47	1.81	-	2.05	[85]
P9	1.75	−5.30	−3.40	1.90	-	4.40	[86]
P10	1.91	−5.25	−3.64	1.62	362	6.12	[83]
P11	1.96	−5.50	−3.54	1.96	-	3.68	[87]
P12	1.96	−5.45	−3.49	1.96	-	5.85	[87]

## Data Availability

All data generated or analysed during this study are included in this published article.

## Supplementary Materials

The following supporting information can be downloaded at: https://www.mdpi.com/article/10.3390/polym17152050/s1, Figure S1: Williamson-Hall Plot (W-H) Plot; Figure S2: Band gap determination for the films using ASF approach; Figure s S3–S7: 1H-NMR spectrum; Figures S8 and S9: GPC graphs; Table S1: Determined energy gap from ASF method; Table S2: Summarized results of the crystal structures [88].
