# Supplementary material for: Two Novel Low-Bandgap Copolymers Based on Indacenodithiophene/Indacenodithienothiophene and Benzothiadiazole Dicarboxylic Imide: Structural Design and DFT/TD-DFT Investigation
Source: Polymers (Basel). 2025 Jul 27;17(15):2050. doi: 10.3390/polym17152050 (PMC12349462; doi:10.3390/polym17152050)
Supplement: Supplementary file 1 [file polymers-17-02050-s001.zip › polymers-3769945-supplementary.pdf]

Two Novel Low Band Gap Copolymers Based on Indacenodi-thiophene/Indacenodithienothiophene and Benzothiadiazole Dicarboxylic Imide: Structural Design and DFT/TD-DFT Investigation

Bakhet A. Alqurashy[1]*, Ary R. Murad[2], Wael H. Alsaedi[3], Bader M. Altayeb[3], Shaaban A. Elroby[4] and Abdesslem Jedidi[4]*

[1] Basic Science and Technologies Department, Applied College, Taibah University, Madina, Saudi Arabia; Bqourasy@taibahu.edu.sa
[2]Department of Chemistry, College of Science, Charmo University, Chamchamal, Sulaymaniyah 46023, Iraq; ary.murad@chu.edu.iq
[3]Department of Chemistry, Faculty of Science, Taibah University, Madina, Saudi Arabia; wsadi@taibahu.edu.sa; Btayeb@taibahu.edu.sa
[4] Department of Chemistry, Faculty of Science, King Abdulaziz University, P.O. Box 80203, Jeddah 21589, Saudi Arabia; skamel@kau.edu.sa; ajedidi@kau.edu.sa

1. Experimental

1.1. Measurements

^{1}H and ^{13}C nuclear magnetic resonance (NMR) spectra were obtained using a Bruker AV 400 (400 MHz) in deuterated $CDCl_3$. ^{1}H-NMR spectra of the polymers were performed on a Bruker Avance III HD 500 (500 MHz) spectrometer at 100˚C, using 1,2-dideutrotetrachloroethance ($C_2D_2Cl_4$) as the solvent. TGA was carried out using a Perkin Elmer TGA-1 thermogravimetric analyzer at a scan rate of 10 ^{o}C min^{-1} under an inert N_2 atmosphere. GPC was conducted on polymer solutions using 1,2,4-trichlorobenzene as the eluent at 140 ^{o}C. Polymer samples were spiked with toluene as an internal reference. The GPC curves were recorded using a Viscotek GPCmax VE2001 GPC solvent/sample module and a Waters 410 Differential Refractometer. The system was calibrated with a series of narrow polystyrene standards (Polymer Laboratories). UV-visible absorption spectra were obtained using a Hitachi U-2010 Double Bean UV/Visible Spectrophotometer. Polymer solutions were prepared in $CHCl_3$ and measured using quartz cuvettes with a 1 cm path length. For thin films measurements, absorption spectra were prepared by drop-casting 1 mg.cm^{-3} polymer solutions in $CHCl_3$ onto quartz substrates. PXRD measurements were recorded on a Bruker D8 advance diffractometer equipped with a CuKα radiation source (1.5418 Å, rated as 1.6 kW). Data were collected over a 2θ range of 2° to 40°. CV measurements were performed using

a Princeton Applied Research Model 263A Potentiostat/Galvanostat. A conventional three-electrode system was employed comprising a platinum disc (the working electrode), Pt wire (the counter electrode) and Ag/Ag^+ (reference electrode). Measurements were conducted in a CH_3CN solution containing 0.1 $mol.dm^{-3}$ tetrabutylammonium perchlorate (TBAP), using polymer films drop-cast from $CHCl_3$ solutions onto the working electrode. Ferrocene (Fc) was used as an internal standard in accordance with IUPAC's recommendations. The energy level of Fc/Fc^+ redox couple taken as –4.8 eV relative to vacuum. The half-wave potential ($E_{1/2}$) of Fc/Fc^+ was found to be 0.08 V versus the Ag/Ag^+ reference electrode. The LUMO energy levels (E_{LUMO}) of the polymers were estimated using the equation: $E_{LUMO}= -(4.8 - E_{1/2,\ Fc,Fc+} + E_{red,\ onset})$. $E_{red,onset}$ is the onset reduction potential versus Ag/Ag+ reference electrode. Similarly, the HOMO energy levels (E_{HOMO}) were calculated using: $E_{HOMO}= -(4.8 - E_{1/2,\ Fc,Fc+} + E_{ox,\ onset})$, where $E_{ox,\ onset}$ is the onset oxidation potential versus Ag/Ag^+ reference electrode.

1.2. Williamson–Hall Analysis of PIDTBDI and PIDTTBDI Copolymers

X-ray diffraction (XRD) was employed to investigate the structural properties of the synthesized donor–acceptor copolymers. To assess crystallite size more comprehensively, we have applied the Williamson–Hall (W–H) method, which considers both size-induced and strain-induced broadening of diffraction peaks. This contrasts with the conventional Scherrer equation, which only accounts for crystallite size effects [1]. The W–H equation is given by:

$$\beta \ cos\theta \ = \ k\lambda/D \ + \ 4 \, \epsilon \ sin\theta$$

where: β is the full width at half maximum (FWHM) in radians, θ is the Bragg angle, λ is the X-ray wavelength, k is the shape factor (taken as 0.94), D is the crystallite size, and ϵ is the microstrain [2]. By plotting $\beta\cos\theta$ against 4 $\sin\theta$, a linear relationship is obtained in which the intercept equals $k\lambda/D$, and the slope yields the strain ϵ [3]. Crystallite size was thus calculated using: $D = k\lambda/\ \mathrm{Intercept}$. This method is particularly effective for polymeric materials exhibiting nanocrystalline domains, as it provides a more complete picture of microstructural behavior. For **PIDTBDI**, three diffraction peaks were identified at 2θ = 4.03°, 9.02°, and 20.28°, with corresponding FWHM values (β) of 1.95, 3.31, and 7.58, respectively. The W–H plot (Figure S1 a)

yielded an intercept of 0.00691, corresponding to a crystallite size of 2.10 nm, indicating small, nanocrystalline domains within a largely amorphous matrix. In contrast, **PIDTTBDI** exhibited peaks at 2θ = 4.39°, 8.31°, and 20.48°, with β values of 1.13, 2.95, and 9.71, respectively. The W–H plot (Figure S1 b) gave an intercept of 0.02312, yielding a larger crystallite size of 6.26 nm, indicative of more ordered packing. These results suggest that **PIDTTBDI** exhibits greater crystallinity than **PIDTBDI**, likely due to structural differences. The larger crystallite size of **PIDTTBDI** may result from its more rigid and planar backbone or stronger π–π interactions, promoting better molecular ordering. Such differences in crystallinity and microstrain can significantly influence the optical, mechanical, and charge transport properties of these polymers, directly impacting their performance in optoelectronic devices [4].

Table S2. Summarized the results crystal structures of **PIDTBDI** and **PIDTTBDI**.

Sample	β	2θ	k	λ	$4sin\theta$	$\beta cos\theta$	intercept	D
PIDTBDI	1.94967	4.0352	0.94	0.15406	0.140825943	0.0340070	0.00691	2.095751
	3.31006	9.0233	0.94	0.15406	0.314647193	0.057592433		
	7.5777	20.2765	0.94	0.15406	0.70409572	0.130190758		
PIDTTBDI	β	2θ	k	λ	$4sin\theta$	$\beta cos\theta$	intercept	D
	1.12975	4.3943	0.94	1.5406	0.153352415	0.019703361	0.02312	6.263685
	2.95159	8.305	0.94	1.5406	0.289645468	0.051379729		
	9.71462	20.476	0.94	1.5406	0.710949778	0.166852487		

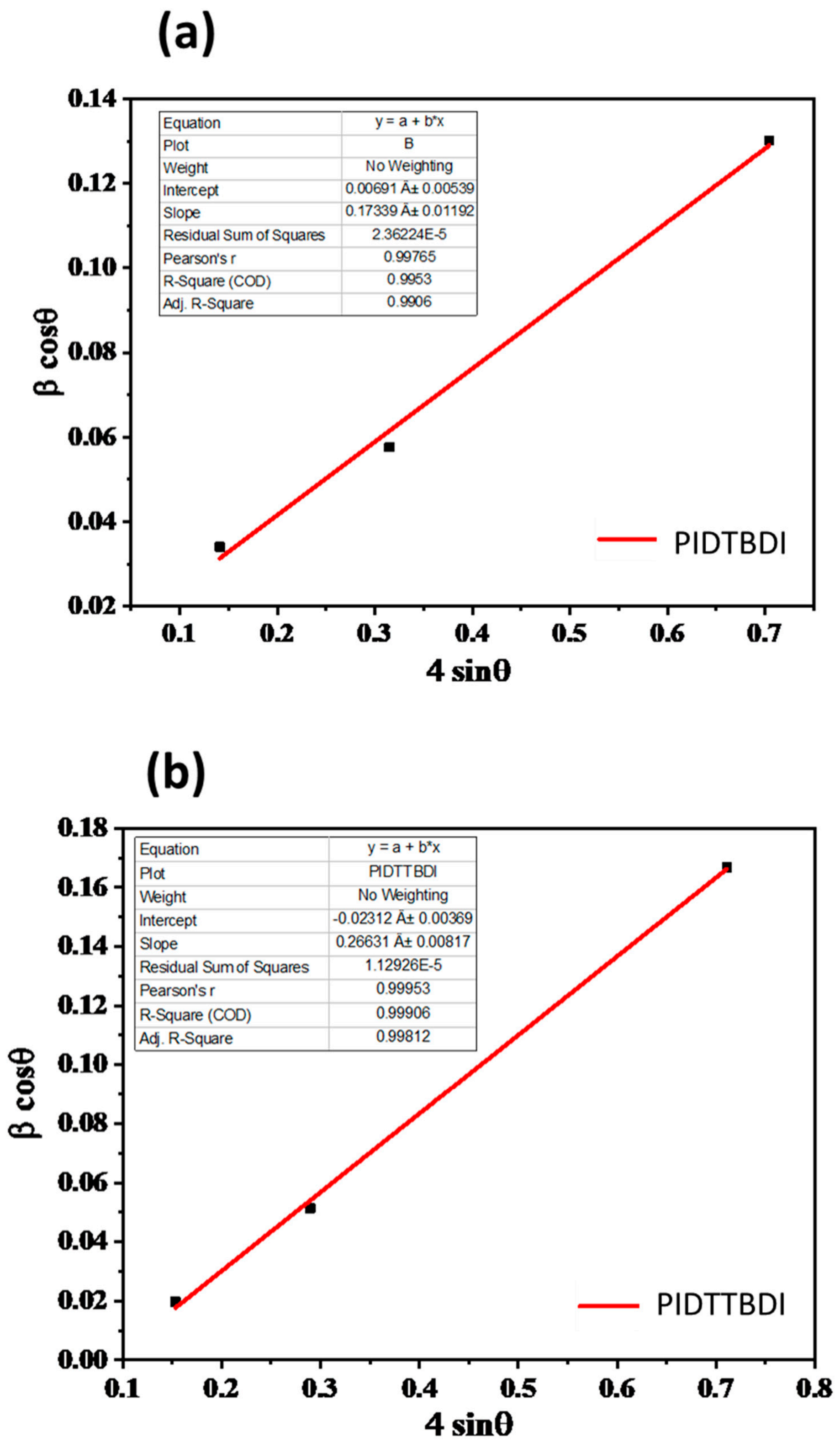

Figure S1. Williamson-Hall Plot (W-H) Plot of **PIDTBDI** (a) and **PIDTTBDI** (b)

1.3. Optical Band Gap Determination by the Absorption Spectrum Fitting (ASF) Method

The nature of electronic transitions in the synthesized polymer films was investigated using the absorption spectrum fitting (ASF) method, which is derived from Tauc's model [5]. The optical band gap (Eg) is associated with transitions from the valence band (VB) to the conduction band (CB) and can be estimated from the UV–Vis absorption spectra. The absorption coefficient (α) is calculated as: $\alpha = 2.303\,A/d$, where A is the absorbance and d is the film thickness [6]. The relationship between the absorption coefficient and the optical band gap is described by Tauc's equation: $(\alpha h v) = B\,(hv - E_g)^{\gamma}$ [5]. Where *B* is a material-dependent constant, *hv* is the photon energy, and *γ* is an index that indicates the nature of the electronic transition: γ = 1/2: allowed direct transition, γ = 3/2: forbidden direct transition, γ = 2: allowed indirect transition, and γ = 3: forbidden indirect transition. Based on Equations (a) and (b), the ASF method reformulates the Tauc equation in terms of wavelength (λ), generating a thickness-independent equation 1:

$$\frac{A(\lambda)}{\lambda} = B_1\,(\frac{1}{\lambda} - \frac{1}{\lambda_g})^{\gamma} \qquad (1)$$

In this equation, B_1 is a constant and λg corresponds to the wavelength at the absorption edge. The optical band gap E_g can be extracted from λg using the relation: $E_g = hc / \lambda g$. To determine E_g, a plot of $(A/\lambda)^{1/\gamma}$ vs. $(1/\lambda)$ is extrapolated to the intercept at $(A/\lambda)^{1/\gamma} = 0$. This graphical method identifies the absorption edge and reveals the nature of the transition. Figure S2 illustrates the ASF plots for the studied films, and the derived Eg values are listed in Table S1. The results obtained from ASF are in good agreement with those estimated using conventional Tauc plots. The analysis suggests that the dominant transitions in films are of the direct allowed type. The ASF method provides a reliable and straightforward approach to analyze the optical transitions in thin films without requiring knowledge of film thickness, making it particularly useful for evaluating solution-processed materials.

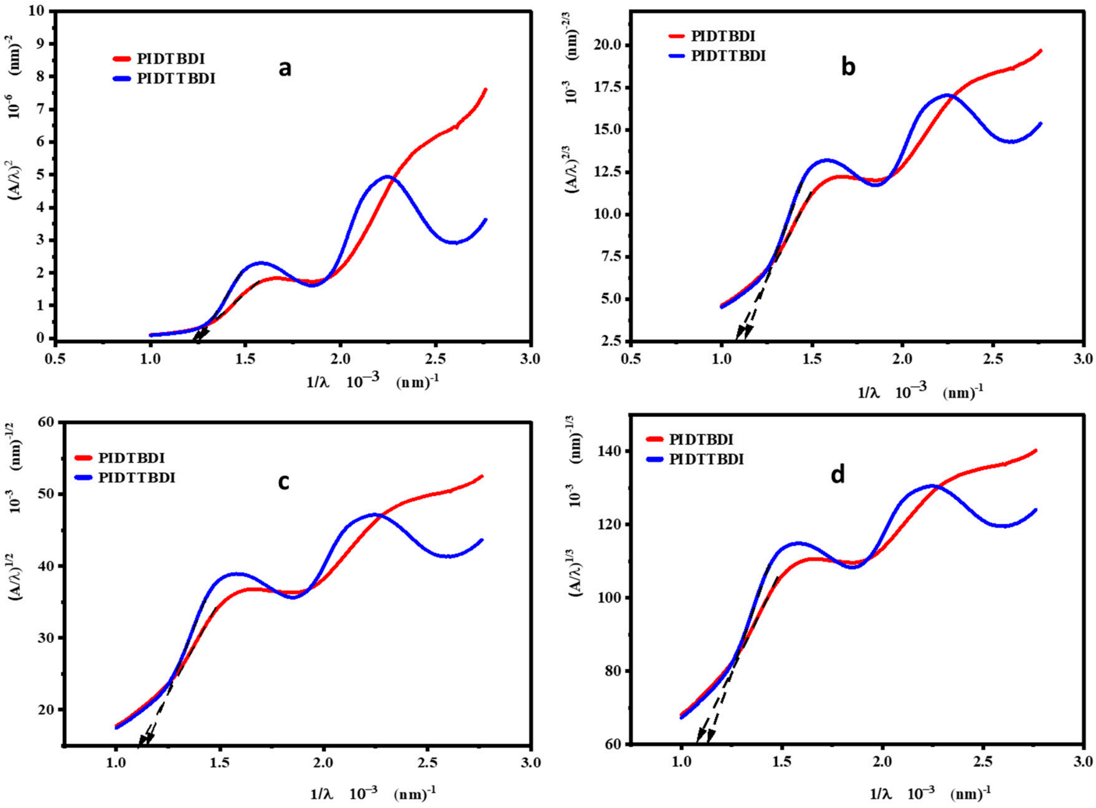

Figure S2. Band gap determination for the films using ASF approach for (a)ϒ = 1/2, (b) ϒ = 3/2 (c), ϒ = 2, and (d) ϒ = 3.

Table S1: Determined energy gap for **PIDTBDI** and **PIDTTBDI** from ASF method.

Sample	Direct allowed	Direct Forbidden	Indirect allowed	Indirect Forbidden
**PIDTBDI**	1.5166125	1.33014375	1.3674375	1.33014375
**PIDTTBDI**	1.55390625	1.3923	1.4171625	1.3923

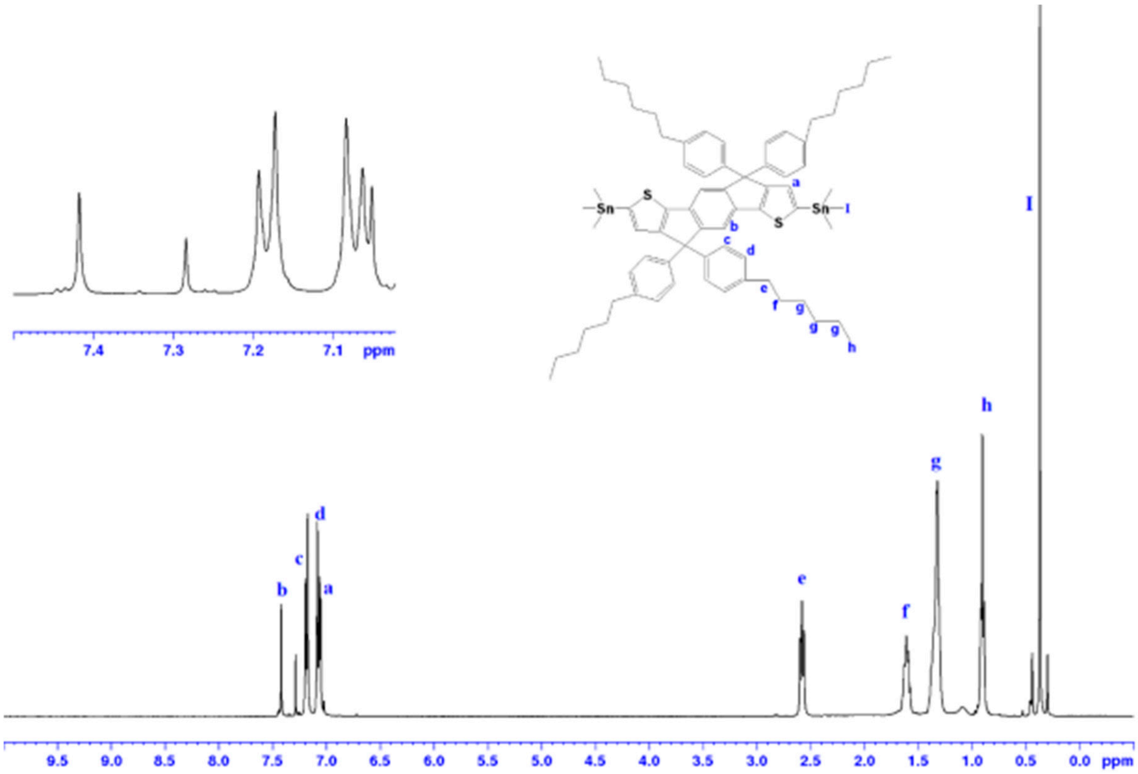

Figure S3. ^{1}H-NMR spectrum of **M1** in $CDCl_3$.

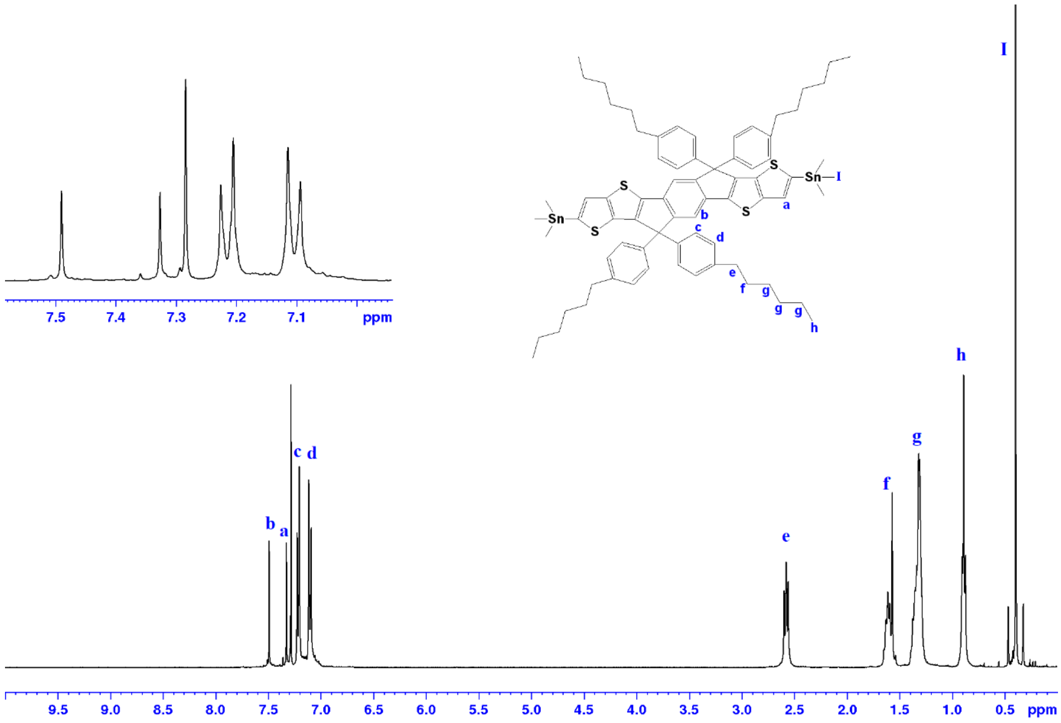

Figure S4. ^{1}H-NMR spectrum of **M2** in $CDCl_3$.

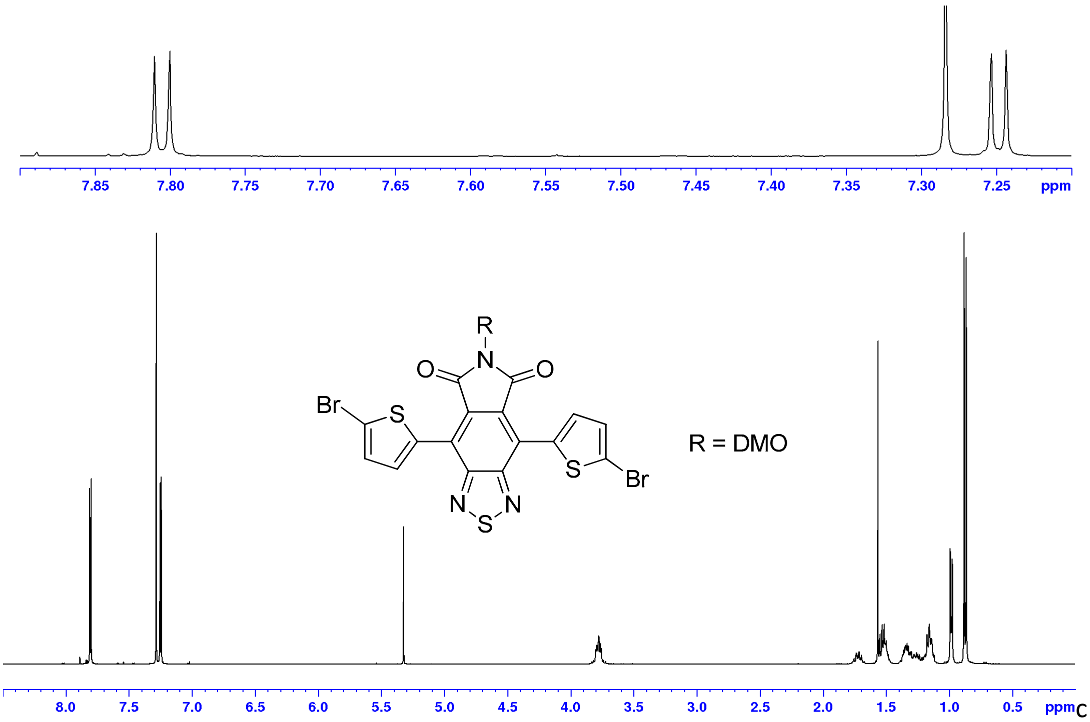

Figure S5. ^{1}H-NMR spectrum of **M3** in $CDCl_3$.

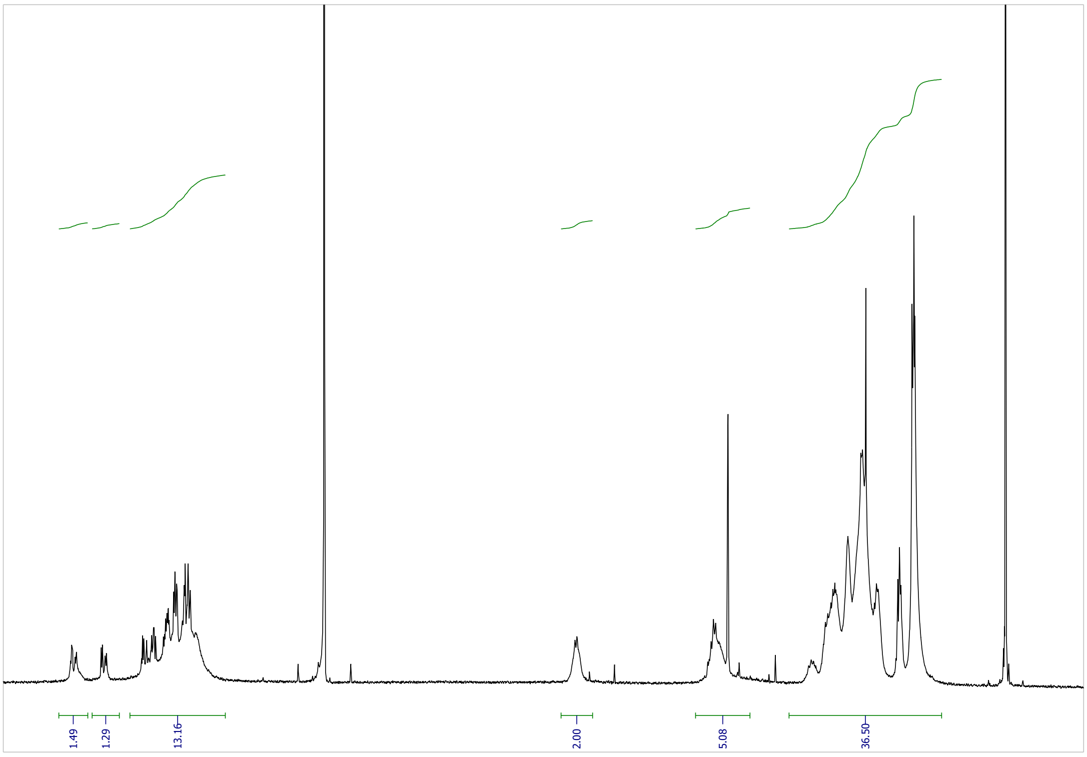

Figure S6. ^{1}H-NMR spectrum of **PIDTBDI** in $C_2D_2Cl_4$.

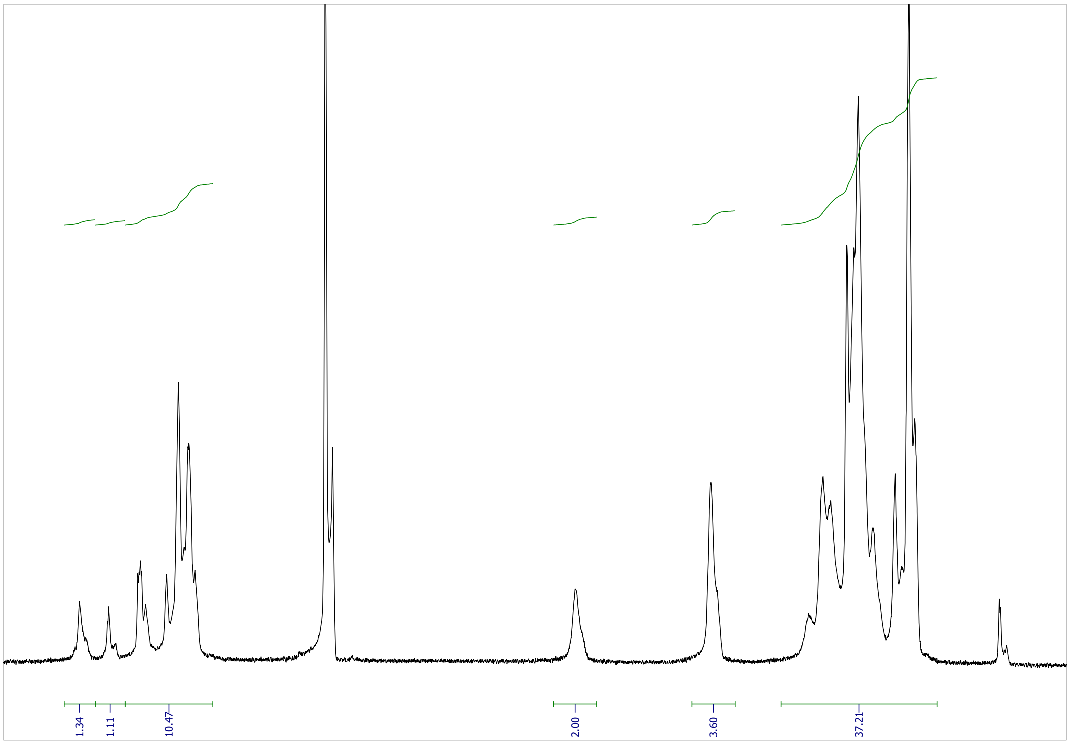

Figure S7. ^{1}H-NMR spectrum of **PIDTTBDI** in $C_2D_2Cl_4$.

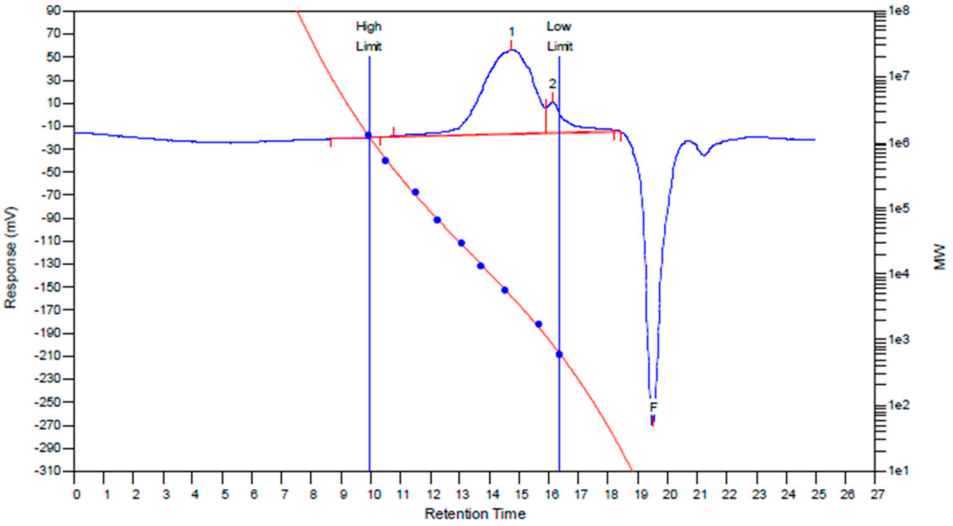

MW Averages

Peak No	Mp	Mn	Mw	Mz	Mz+1	Mv	PD
1	4516	4334	10673	67825	215669	8581	2.46262

Figure S8. GPC graph of **PIDTBDI**.

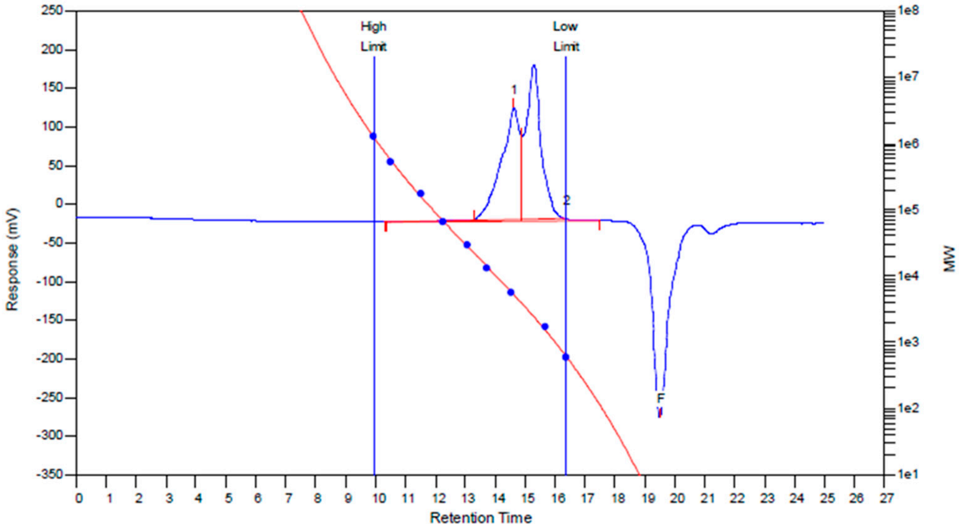

MW Averages

Peak No	Mp	Mn	Mw	Mz	Mz+1	Mv	PD
1	5247	6236	7149	8459	10147	6983	1.14641

Figure S9. GPC graph of **PIDTTBDI**.

References:

1. Bindu, P. and S. Thomas, *Estimation of lattice strain in ZnO nanoparticles: X-ray peak profile analysis.* Journal of Theoretical and Applied Physics, 2014. **8**(4): p. 123-134.
2. Akl, A.S. and M. Elhadi, *Estimation of crystallite size, lattice parameter, internal strain and crystal impurification of nanocrystalline Al3Ni20Bx alloy by Williamson-Hall method.* J. Ovonic Res., 2020. **16**(5): p. 323-335.
3. Irfan, H., M. Racik K, and S. Anand, *Microstructural evaluation of CoAl2O4 nanoparticles by Williamson–Hall and size–strain plot methods.* Journal of Asian Ceramic Societies, 2018. **6**(1): p. 54-62.
4. Madivalappa, S., et al., *Insights and perspectives on PVDF/MgO NCs films: Structural and optical properties for optoelectronic device applications.* Results in Chemistry, 2024. **11**: p. 101764.
5. J. Tauc, Amorphous and Liquid Semiconductors, Plenum, New York, 1974, pp. 159–220.
6. Alsoghier HM, Selim MA, Salman HM, Rageh HM, Santos MA, Ibrahim SA, Dongol M, Soga T, Abuelwafa AA (2018) NMR spectroscopic, linear and non-linear optical properties of 1,3-benzothiazol-2-yl-(phenylhydrazono) acetonitrile (BTPA) azo dye. J Mole Struct 1179:315 ‑ 324
